# The Role of Arachidonic and Linoleic Acid Derivatives in Pathological Pregnancies and the Human Reproduction Process

**DOI:** 10.3390/ijms21249628

**Published:** 2020-12-17

**Authors:** Małgorzata Szczuko, Justyna Kikut, Natalia Komorniak, Jacek Bilicki, Zbigniew Celewicz, Maciej Ziętek

**Affiliations:** 1 Department of Human Nutrition and Metabolomics, Pomeranian Medical University, Broniewskiego 24 Street, 71-460 Szczecin, Poland; justyna.kikut@pum.edu.pl (J.K.); natalia.komorniak@pum.edu.pl (N.K.); 2 Department of Neurosurgery and Pediatric Neurosurgery, Pomeranian Medical University, Unii Lubelskiej 1 Street, 71-252 Szczecin, Poland; jacek.bilicki@gmail.com; 3 Department of Perinatology, Obstetrics and Gynecology, Pomeranian Medical University, Siedlecka 2 Street, 72-010 Police, Poland; sekr.perinat@spsk1.szn.pl (Z.C.); maciej.zietek@pum.edu.pl (M.Z.)

**Keywords:** pregnancy disorders, inflammation, HETE, HODE, arachidonic acid, polycystic ovarian syndrome, endometriosis, preeclampsia, gestational diabetes mellitus

## Abstract

The aim of the available literature review was to focus on the role of the proinflammatory mediators of AA and LA derivatives in pathological conditions related to reproduction and pregnancy. Arachidonic (AA) and linoleic acid (LA) derivatives play important roles in human fertility and the course of pathological pregnancies. Recent studies have demonstrated that uncontrolled inflammation has a significant impact on reproduction, spermatogenesis, endometriosis, polycystic ovary syndrome (PCOS) genesis, implantation, pregnancy and labor. In addition, cyclooxygenase-mediated prostaglandins and AA metabolite levels are higher in women’s ovarian tissue when suffering from PCOS. It has been demonstrated that abnormal cyclooxygenase-2 (COX-2) levels are associated with ovulation failure, infertility, and implantation disorders and the increase in 9-HODE/13-HODE was a feature recognized in PCOS patients. Maintaining inflammation without neutrophil participation allows pregnant women to tolerate the fetus, while excessive inflammatory activation may lead to miscarriages and other pathological complications in pregnancies. Additionally AA and LA derivatives play an important role in pregnancy pathologies, e.g., gestational diabetes mellitus, preeclampsia (PE), and fetal growth, among others. The pathogenesis of PE and other pathological states in pregnancy involving eicosanoids have not been fully identified. A significant expression of 15-LOX-1,2 was found in women with PE, leading to an increase in the synthesis of AA and LA derivatives, such as hydroxyeicozatetraenoic acids (HETE) and hydroxyoctadecadiene acids (HODE). Synthesis of the metabolites 5-, 8-, 12-, and 15-HETE increased in the placenta, while 20-HETE increased only in umbilical cord blood in women with preeclampsia compared to normal pregnancies. In obese women with gestational diabetes mellitus (GDM) an increase in epoxygenase products in the cytochrome P450 (CYP) and the level of 20-HETE associated with the occurrence of insulin resistance (IR) were found. In addition, 12- and 20-HETE levels were associated with arterial vasoconstriction and epoxyeicosatrienoic acids (EETs) with arterial vasodilatation and uterine relaxation. Furthermore, higher levels of 5- and 15-HETE were associated with premature labor. By analyzing the influence of free fatty acids (FFA) and their derivatives on male reproduction, it was found that an increase in the AA in semen reduces its amount and the ratio of omega-6 to omega-3 fatty acids showed higher values in infertile men compared to the fertile control group. There are several studies on the role of HETE/HODE in relation to male fertility. 15-Hydroperoxyeicosatetraenoic acid may affect the integrity of the membrane and sperm function. Moreover, the incubation of sperm with physiologically low levels of prostaglandins (PGE2/PGF2α) improves the functionality of human sperm. Undoubtedly, these problems are still insufficiently understood and require further research. However, HETE and HODE could serve as predictive and diagnostic biomarkers for pregnancy pathologies (especially in women with risk factors for overweight and obesity). Such knowledge may be helpful in finding new treatment strategies for infertility and the course of high-risk pregnancies.

## 1. Introduction

The purpose of this review was to systematize information on the involvement of proinflammatory mediators of AA and LA derivatives in pathological conditions related to reproduction and pregnancy. Knowing this can help find new strategies for infertility treatment and may prove useful in planning the course of pregnancies.

The course of inflammation is associated with the activation of the coagulation system, increased perfusion and vascular permeability, as well as the production of inflammatory mediators, including arachidonic acid (AA) and linoleic acid (LA) derivatives. Inflammation is associated with the normal course of menstrual cycles, the embryo implantation process, the course of pregnancy, and childbirth. In physiological conditions it is important not only for the proper course of the entire menstrual cycle, but also for the creation of an implantation window enabling the correct nesting of the embryo. The induction of labor is connected with the appearance of neutrophils and macrophages. AA is the most abundant fatty acid that is the main precursor to proinflammatory eicosanoids in macrophages. In order to maintain balance, it is important that proinflammatory signals are regulated by anti-inflammatory mediators [1]. The authors described the aspects of the role of AA and LA derivatives in the course of properly running processes in another article [2]. However, in this paper, they focus on pathological processes.

In menstrual cycles, which run in four phases, the pathological course of inflammation may be accompanied by endometriosis and polycystic ovary syndrome (PCOS). Both pathologies will be discussed separately, later in this article. Although maintaining inflammation during pregnancy enables the mother to tolerate the fetus, excessive activation may lead to adverse effects. Common pregnancy pathologies related to inflammation reactions include miscarriage, preeclampsia (PE), gestational diabetes mellitus, premature labor and low fetal birth weight [3]. It has been shown that both free fatty acids (FFA) and their derivatives take part in the inflammatory reaction accompanying the course of pregnancy [4,5]. The inflammation plays a key role in the normal pregnancy course, uterine muscle contractile activity stimulation, cervical ripening and labor. Pregnancy, as a state of immunomodulation, is distinguished by an early period, i.e., implantation, a middle period and a late one, i.e., delivery. Both are considered to be proinflammatory responses [1,6].

### Physiologic Mechanisms of Polyunsaturated Fatty Acids

Polyunsaturated fatty acids (PUFAs) are divided into omega-3 (n-3) fractions (with the main synthesis pathway from α-linolenic acid) and omega-6 (n-6), which is synthesized from linoleic acid (LA) [7]. Linoleic acid can be metabolized to n-6 via desaturase, resulting via the biosynthesis of gamma-linolenic acid (GLA), dihomo-gamma-linolenic acid (DGLA), and finally AA [4]. The largest amount of arachidonic acid (AA), however, is found in phospholipid membranes, competing with n-3 acids for metabolism and with their products for receptors [4,5]. 

**Figure 1:**
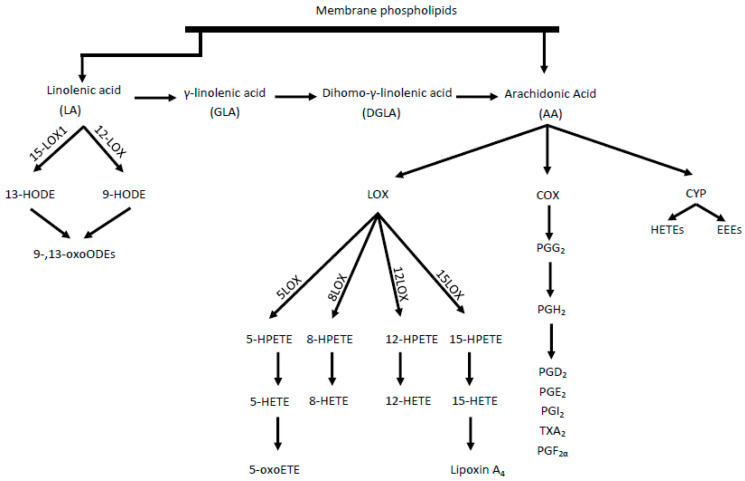
Synthesis of proinflammatory mediators from linoleic acid (LA) and arachidonic acid (AA). LOX—lipoxygenase, HETE—hydroperoxyeicosatetraenoic acid, HPETE—Hydroperoxyeicosatetraenoic *acid*, HODE—hydroxyoctadecadiene acids, COX—cyclooxygenase, CYP—cytochrome P450, HETEs—hydroxyeicosatetraenoic acids, EETs—epoxyeicosatrienoic acids, PG—prostaglandins, TX—thromboxane.

Under the influence of cyclooxygenase (COX) enzymes, AA is transformed into prostaglandins (PG), thromboxanes, and leukotrienes. Eicosanoids, mainly epoxyeicosatrienoic acids (EETs) and 20-hydroxyeicosatetraenoic acid (20-HETE), are produced throughout cytochrome P450 (CYP) enzyme activity [8]. However, as a result of AA metabolism mediated by lipooxygenase (LOX) enzymes, including 5-, 8-, 12-, and 15-LOX, from which, in turn, HPETE acids are adequately produced, followed by HETE and oxo-ETE, shown in Figure 1 [9]. A strong chemoattractant for basophils, eosinophils, monocytes and neutrophils is 5-oxoETE, 12 HETE. Moreover, 15 HETE stimulates mitogenesis of endothelial cells, increases the activity of granulocytes and lymphocytes.

Two isoforms of the COX enzyme (COX-1 and COX-2) are distinguished. COX-1 participates in basic physiological functions, such as platelet aggregation control, while COX-2 mainly takes part in inflammation and pathophysiological processes [10]. LA acid metabolism (by LOX enzymes) leads to the formation of hydroxyoctadecadienoic acids (HODEs), i.e., 9- and 13-HODE and subsequently oxo-HODE [4]. Both 13-HODE and 9-HODE should be regarded as atherosclerosis biomarkers. Eicosanoids are also formed from the n-6 pathway, which, together with other oxidized fatty acids metabolites, are called oxylipins. The oxylipin presence is correlated with the occurrence of inflammation and the main pathways for their synthesis, comparable with AA, are the COX, LOX, and CYP pathways [11]. Some CYP polymorphisms are associated with many diseases, including cancer and cardiovascular diseases. The CYP4A10 and CYPA12 isoforms are enzymes associated with hepatic and renal biosynthesis of atherosclerotic 20-HETE. In contrast, CYP2J2 is responsible for the formation of EET in extrahepatic tissues, including the heart, kidneys, lungs, and intestines in humans [12].

## 2. Participation of AA Metabolites in Fertility

### 2.1. Endometriosis

**Figure 2:**
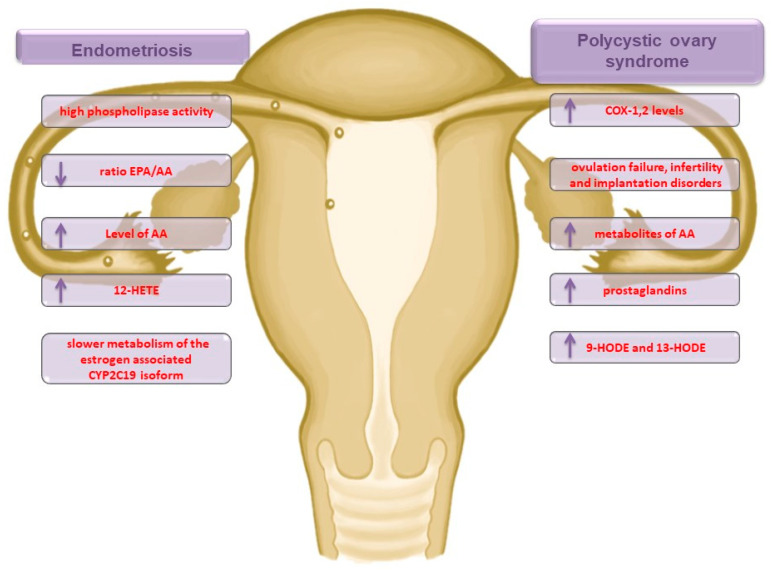
Participation of AA metabolites in pathological conditions of pregnancy—endometriosis and polycystic ovary syndrome; PCOS: polycystic ovary syndrome; COX: cyclooxygenase; EPA: eicosapentaenoic acid; AA: arachidonic acid; CYP2C19: cytochrome isoform; HODE: hydroxyoctadecadiene acids.

Endometriosis is a gynecological disorder that affects between 7 and 10% of women of reproductive age [13]. The primary symptom of endometriosis is pelvic pain, often associated with dysmenorrhea, significantly lowering patients’ quality of life [13]. The exact cause of the disease is still unknown, although its pathogenesis may be related to genetic, hormonal, immunological, and inflammatory factors [14]. Khanaki et al. [15] showed that a decreased eicosapentaenoic acid (EPA) to AA ratio (EPA/AA) is directly correlated with the severity of disease, and, in addition, Li et al. [16] demonstrated that the AA concentration was significantly higher in women suffering from endometriosis than in women from a control group. Moreover, there was an 82% lower incidence rate of endometriosis among patients with higher EPA blood levels when compared to those with low EPA levels [17]. This means that low EPA levels and high phospholipase activity may play an important role as a cause of endometriosis. In some studies analyzing the participation of HETE in endometriosis pathogenesis, it has been found that inflammation expressed by the presence of 12-HETE differs in a manner relative to the endometrial cell location. The occurrence of endometrial cells in the peritoneum is associated with a significantly higher serum level of 12-HETE in comparison with endometrial foci located in ovarian tissue [18]. In addition, 12- and 15-HETE are responsible for activating the capsaicin-sensitive vanilloid receptor (VR1), which is correlated with inflammatory pain signaling [19]. In contrast, endometriosis was associated with slower metabolism of the estrogen-associated CYP2C19 isoform [20]. As no more data regarding the inflammation state expressed via the presence of HETE and HODE in endometriosis have been found, further research is required. Moreover, EPA levels and the EPA/AA ratio play a key role in endometriosis. In addition, there appears to be an intensified pathway for the synthesis of 12-HETE mediators and for women with pain, the path for 15-HETE synthesis (Figure 2). The review of Bulun et al. [21] showed the important role of COX-2 in the course of endometriosis. Higher expression of COX-2 in the endometrium and higher levels in endometrial stromal cells were observed in the study.

### 2.2. Polycystic Ovary Syndrome

Polycystic ovary syndrome (PCOS) is one of the most common endocrinopathic complications for women of childbearing potential. The disease is multifactorial and is often associated with generalized body inflammation, and thus an increased risk of infertility, insulin resistance (IR), and obesity, as well as cardiovascular disorders [22]. It has been demonstrated that abnormal COX-2 levels are associated with ovulation failure, infertility, and implantation disorders [23]. In a rat model study, it was shown that the levels of PG and metabolites of AA (via the COX pathway) were significantly higher in the ovarian tissue of the PCOS group than in that of the healthy control group [24]. In turn, human studies have shown a significant increase in the 9-HODE/13-HODE serum level among patients with PCOS [25].

It proves very important, because the combination therapy of pioglitazone/flutamide/metformin leads to a significant reduction in the levels of 9-HODE, 13-HODE, and derivatives of LA, which in turn translates into a reduction in the inflammation state and related complications, including a higher risk of atherosclerosis [26,27]. The weight of women suffering from PCOS is also an important factor affecting inflammation intensity, with significantly lower 5-HETE and 12-HETE concentrations among underweight patients compared to obese patients [28]. Very interesting conclusions were reached by Szczuko et al. [29]. The authors have shown that women suffering from PCOS who have experienced pharmacotherapy have a significantly lower concentration of inflammatory mediators (5-, 12-, 15-, and 16-HETE and 9- and 13-HODE) in their blood when compared to potentially healthy women in a control group. In addition, after applying nutritional intervention in the form of reducing diet, following a low glycemic index, a significant increase in the concentration of inflammatory mediators among people with PCOS has been observed. The authors suggested that this may be related to the activity intensification or the activation of suppressed repair processes, which previously (with a low mono- and polyunsaturated and rich saturated fatty acid diet) were inefficient. In summary, in the pathophysiology of PCOS, the LOX pathways for 5-, 12-, and 15-HETE synthesis are activated. In addition, the synthesis enhancement is noticeable for the COX pathway, which leads to high serum levels of PG and 9- and 13-HODE, which can be used as a target in PCOS therapy (Figure 2).

### 2.3. Long-Term Health Problems

With regard to the disorders described above, their long-term consequences can be expected. Endometriosis may affect the occurrence of endometrial cancer, which is one of the most common cancers of the female genital organs. It has been identified that it is excessive body weight and excessive endometrium by estrogen that are the most important factors of this type of cancer [30]. The studies have shown a positive effect of dietary omega 3 fatty acids on growth inhibition of endometrial cancer cells in vivo and their ability to prevent metastases [30]. COX-2 is overexpressed in the case of proliferation and endometrial cancer. It also appears that increased expression of COX-2 may identify the degree of aggression of endometrial cancer [31]. In addition, it has been shown that increased levels of ALOX5 and decreased levels of 15-hydroxyprostaglandin dehydrogenase (HPGD) are associated with adverse prognosis in endometrial cancer [32]. Moreover, PCOS increases the risk of hyperinsulinemia and cardiovascular disease in the long term and may increase the risk of endometrial cancer through undiagnosed ovulation [33].

### 2.4. Male Reproduction

The nature of subfertility in the male is as complex as in female patients. The idea that absolute sperm count determines fertility is wrong, because it is their functional competences like the induction of acrosomal exocytosis and connection of the sperm with the oocyte that condition fertilization. Male infertility is mainly associated with sperm dysfunction, and more specifically, reduced sperm motility [34,35].

The influence of BMI on the composition of the fatty acids of sperm has been demonstrated. The body mass index itself is negatively related to the total number and viability of sperm. High body BMI is negatively associated with semen quality, while BMI was negatively related to the level of docosahexaenoic acid (DHA) in the sperm [35]. DHA removal from the sperm membranes during the process of sperm maturation would be expected to lead to a decrease in membrane fluidity [36]. The meta-analysis showed that supplementation of infertile men with omega-3 (DHA and EPA) improved sperm motility and the concentration of DHA in semen plasma [37,38]. Moreover, a male semen defect was found with a simultaneous increase in the excess of PUFA, including AA in sperm, which promoted excessive production of ROS and lipid peroxidation [36]. In the study by Oborna et al., it was noted that oxidative stress is associated with increased lipid peroxidation, which affects changes in the profile of fatty acids in the semen of normozoospermic men (patients whose semen has normal parameters) [39]. In turn, the Tavilani et al. study showed a higher PUFA/SFA ratio in the semen of normozoospermic men compared to astenozoospermic men (i.e., the semen had reduced sperm motility) [40]. It has been observed also that such a PUFA/SFA ratio may be associated with reduced sperm motility [40]. Studies involving 82 men showed that the ratio of n-6 to n-3 acids measured in sera showed higher values in infertile men compared to the fertile control group [41]. Higher levels of n-6 in sera are correlated with reduced sperm motility, as well as a reduced sperm count [41].

There are few reports on the role of HETE/HODE arachidonic acid derivatives in relation to male fertility. AA and its derivatives derived from the lipoxygenase pathway are associated with the steroidogenesis process by affecting cholesterol transport [42]. Metabolites from the LOX pathway stimulate steroidogenesis. It has been shown that mouse Leydig cell stimulation increases the levels of 5-HPETE and 5-HETE [43]. On the other hand, the inhibition of 5-LOX activity limits the steroidogenesis process [44]. A study by Olive et al. confirmed the activity of 15(S)-lipooxygenase in purified proteosomes of human semen that bind to germ cells [45]. In another study on bulls, 15(S)-LOX was found to be involved in the acrosome reaction of sperm [46]. Olive et al. also suggested that 15-hydroperoxyeicosatetraenoic acid may affect the integrity of the membrane and consequently the function of sperm [45]. Physiologically speaking, vesicular discharge contains PG.

Once the follicular secretion is combined with the fluid excreted by the prostate and ejaculatory ducts of the vesicles, PGs increase the uterine contractile activity thus facilitating the mixing of semen with fallopian tube fluid [47].

It has already been shown that human semen is characterized by a significant amount of prostaglandin E, which has a positive effect on sperm motility and their ability to penetrate [45]. However, research on assisted reproduction methods proved that the incubation of sperm with physiologically low levels of PGE2/PGF2α improves the functionality of human semen [48].

A self-protective mechanism against inflammation and oxidative stress is based on PG action. The PGs regulate also the recruitment of energy substrates to sperm metabolic needs via peroxisome proliferator-activated receptor gamma [49].

The above data suggest that the composition of fatty acids is extremely important for male fertility. In addition, fertility problems may cause excessive production of reactive oxygen species, which also greatly interfere with semen function. To complete the picture of inflammation, it would be worthwhile exploring the participation of HETE/HODE in male infertility. Male fertility may be affected by induction of inflammatory biomarkers in prostatic diseases leading to decreased sperm cell survival, dysfunction and abnormalities, e.g., chronic leukocytospermia. The novel inflammation biomarkers such as TLR-4, COX-2, and Nrf-2 may play a key role in idiopathic male infertility states [50]. Additionally, the protective effects of antioxidant proteins (superoxide dismutase, catalase, and peroxiredoxins) are decreased in chronic inflammatory conditions and excess generation of reactive oxygen species is known to be related with sperm damage [50]. The sperm abnormalities and functional deficits have been also demonstrated in varicocele and DM.

## 3. Participation of AA Metabolites in Pregnancy

### 3.1. The Implantation and Fetal Growth

The implantation consists of penetrating the blastocyst into the uterine mucosa, “damaging” the endometrium and replacing the endothelium, i.e., the uterine blood vessels with trophoblasts. To achieve successful implantation, the uterus must undergo structural and functional remodeling. The whole remodeling process requires an inflammatory environment to repair the uterine epithelium and remove cellular debris [6]. An influx of immune cells is observed, such as macrophages, natural killer cells, and dendritic cells, which penetrate the decidua and accumulate around trophoblasts [13]. The absence of immune cells would lead to implantation failure, abnormal placenta development, and consequently termination of the pregnancy [51]. In this “physiological” inflammation state, however, no influx of neutrophils (which are known to be recruited first to the site of infection) is observed, which suggests the presence of a self-limiting inflammatory process, thereby preventing full immune response development [15]. Such modification probably leads to the stabilization of placental accommodation. The occurrence of inflammation without implanted embryo destruction is referred to as an inflammation paradox [52]. In contrast, the middle time of a pregnancy is referred to as an anti-inflammatory period [6].

In the next phase of pregnancy, the mother, placenta, and intensely growing fetus remain in symbiotic relationships. A phenomenon called placental biomagnification is observed when both the n-3 and n-6 fatty acids are actively transported through the placenta and are incorporated into fetal tissues, erythrocytes, and nervous system tissues [53].

In the last stage of pregnancy, the appearance of an inflammation state is required once again, during which the influx of immune cells in the myometrial layer is observed. Thus, uterine contractions and childbirth with progressive placenta detachment from the uterine wall are triggered [6]. AA metabolites are thought to have a modulating effect on uterine contractility [54]. Although maintaining an inflammation state in pregnancy enables the mother to tolerate the fetus, excessive activation may lead to pathological pregnancy complications, such as premature labor and fetal growth restriction [55].

It is well known that immune responses are indispensable to promote healthy pregnancy. A physiologic regulation of the innate immune responses during pregnancy serves as a prevention of the fetal allograft rejection. The cytokine production plays a crucial role, where interleukin 2, interferon gamma, IL-10, IL-17 and chemokines are essential. The maternal inflammation system throughout pregnancy is directly dependent on the balance of pro- and anti-inflammatory cytokine ratio. In normal pregnancy circumstances, the Th1/Th2 activity balance is strongly shifted toward Th2 activity. Th1 cells produce IL-1, IL-2, IL-6, IL-12, IL-15, IL-18, IFN-g, and TNF-a while Th2-cells are responsible for IL-4, IL-5, IL-10, IL-13, and GM-CSF production. In some pregnancy pathologies, the expression of proinflammatory (IL-6, TNF-α) and anti-inflammatory (IL-4, IL-10) cytokines is altered. This Th1 predominance state leads to the intensification of inflammatory cytokine production, which is also associated with spontaneous abortion and preterm deliveries. An inflammatory reaction with participation of arachidonic and linoleic acid derivatives (HETEs and HODEs) is normally present in an uneventful pregnancy [2]. In pathology, arachidonic acid transformation via the COX pathway initiates the formation of prostaglandins and thromboxane which are related to fetal growth retardation.

It is possible that other than specific transcription relationships are involved in biochemical, cellular, and signal transduction pathways. As regulatory gene members are interconnected, the regulation may be present at the transcriptional or protein−protein and metabolite−protein level. The biochemical pathway conception of the gene network and its regulative role during pregnancy is still not explicitly presented in literature and there is insufficient data on this topic.

### 3.2. Obesity and the Development of Gestational Diabetes Mellitus

Obesity before pregnancy presents a predisposition to gestational diabetes mellitus (GDM) [56]. GDM is one of the most common complications during pregnancy. It is estimated that GDM affects as many as 18% of women in the world population. Many factors contribute to its occurrence, e.g., excessive body weight, age of the mother and her diet [57]. GDM is defined as abnormal carbohydrate tolerance detected for the first time during pregnancy, which normalizes after birth [58]. Excessive body weight is characterized by an increase in the amount of proinflammatory oxylipids, which in turn leads to the formation of chronic inflammation state [56]. When compared to normal body weight patients, obese patients show a higher concentration of metabolites such as 5- and 15-HETE [59]. It has also been suggested that obesity may adversely affect the fetal lipid profile, especially in the late stage of pregnancy and may be related with other pathologies during pregnancy [60]. An increase in the amount of CYP epoxygenase products, i.e., 9-, 10-, 12-, and 13-epoxyctadecenoic acid, has been observed as well [59]. Another study has shown a higher concentration of 20-HETE in the sera of obese women compared to a group with a normal body weight. Strong adipogenic 20-HETE activity has also been observed [61]. Another study has demonstrated an increased 20-HETE concentration in both the urine and plasma of a group of patients with metabolic syndrome, compared to a control group [62].

In addition, the level of 20-HETE in blood is significantly correlated with body mass index (BMI), where the higher the BMI is, the higher the concentration of this metabolite [63]. In another study conducted on mice fed a high-fat diet, increased levels of 20-HETE were observed in both peripheral circulation and adipose tissue. In addition, a correlation with impaired insulin signaling was demonstrated. It leads to a conclusion that 20-HETE has a significant impact on obesity induced by a high-fat diet, impaired insulin signaling, and the occurrence of insulin resistance (IR) [64].

An innovative approach was suggested by Facchinetti et al. through the application of substances such as myo-inositol and D-chiro-inositol used for the treatment of IR, which may complement medical care during GDM treatment [65,66].

**Figure 3:**
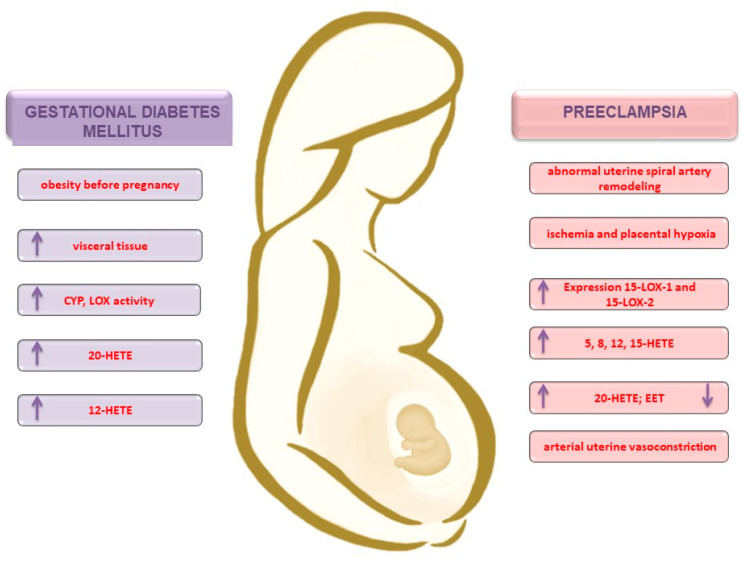
Participation of AA metabolites in pathological conditions of pregnancy—gestational diabetes mellitus and preeclampsia. CYP: cytochrome P450; LOX: lipoxygenase; HETE: hydroperoxyeicosatetraenoic acid; EET: epoxyeicosatrienoic acid.

Studies conducted with obese patients have shown a significant expression of arachidonate 12-LOX in visceral adipose tissue when compared to subcutaneous adipose tissue in both subjects with and without type 2 diabetes mellitus (DM2). In contrast, arachidonate 15-lipoxygenase type A (LOX 15a) expression in SC has been observed in none of the groups, while arachidonate 15-lipoxygenase type B (LOX-15b) increased in both visceral adipose tissue (VAT) groups. A slight increase in 12-HETE concentration was observed in VAT group patients with DM2. The effect of diabetes mellitus on 12-HETE expression depends on the type of adipose tissue. The 12-HETE appears to be an important inflammatory metabolite associated with visceral adipose tissue in DM2 [67]. In the study of Song et al. [68], 15-HETE-induced adipogenesis has been observed in both human and mouse bone marrow stem cells. In addition, there was an increase in lipid accumulation and adipogenesis in the 3T3-L1 mouse (adipocyte-like) cell line (Figure 3).

Diabetes mellitus can be caused by many factors, including previous IR, pancreatic beta cell defects, and an improper diet [69]. Diabetes mellitus is also connected with complications that are dangerous for both the mother and child. These complications include fetal macrosomia, birth injuries, such as infant shoulder dystocia with subsequent brachial plexus palsy or broken bones, and postnatal hypoglycemia [70]. In addition, a study enabling the identification of carbohydrate disorders was performed in the late second trimester of pregnancy, which significantly delayed the possibility of starting treatment to prevent irregularities [71]. The total HETE concentration in the placenta of pregnant women with insulin-dependent diabetes mellitus increased significantly [72]. GDM is also associated with placental hypersecretion of proinflammatory cytokines. In addition, high glucose levels can additionally stimulate proinflammatory cytokines, which affect normal placental development [56]. The syncytiotrophoblast layer is the primary barrier to maternofetal fatty acid transfer through the human placenta, and the fetus is dependent on syncytiotrophoblast-delivered fatty acids. Lipid storage in syncytiotrophoblasts has been observed in obese pregnant women. Palmitoleic fatty acid levels are reduced in syncytiotrophoblasts isolated from maternal obesity-complicated pregnancies, which can lead to IR and persistent mild inflammation in both the mother, placenta, or fetus [73].

In the plasma of diabetic patients, a significantly higher concentration of 12-HETE and 12-HETrE 12-lipoxygenase, as well as levels of 5-HETE and 20-HETE, have been observed [63].

In the Umeno et al. study [74], 10- and 12-(Z,E)-HODE levels were correlated with fasting plasma glucose and the glucose levels measured after the oral glucose tolerance test. Moreover, the 10- and 12-(Z,E)-HODE levels were also correlated with the HbA1c concentration.

In the study of Laffer et al. [75], 20-HETE excretion was observed to be lower in the patient group with hypertension and IR compared to those with hypertension, but not those with insulin sensitivity. This means that the higher the insulin level, the less 20-HETE is excreted in urine. It appears that impaired 20-HETE synthesis induced by an increase in insulin levels may result from phospholipase A2 inhibition, which is an enzyme responsible for the release of AA substrates into cytosols.

A study by Chen et al. [76] of pregnant women with GDM found a positive correlation between palmitic, stearic, AA, and DGLA with the insulin resistance index and C-peptide level. This strongly confirms the impact of saturated fatty acids and AA and DGLA on the occurrence of IR. Similarly, in other studies involving diabetic women, palmitic acid, AA, and total saturated fatty acid plasma levels in the third trimester have been shown to be higher when compared to the control group. In addition, a ratio of n-3 to n-6 metabolites in relation to linoleic acid were higher in the diabetic group [77]. GDM is often associated with prepregnancy obesity, which triggers the LOX and CYP pathways associated, in particular, with the synthesis of 12-HETE and 20-HETE, thus being the most convenient therapeutic intervention site.

### 3.3. Preeclampsia

Preeclampsia (PE) is a serious pregnancy complication which is mostly associated with hypertension and proteinuria, which usually appears after 20 weeks of pregnancy. The pathogenesis of preeclampsia is not fully identified yet. A possible mechanism of PE may be related to the release of placental factors, thereby causing vasoconstriction and ischemia. It is known from the literature that PE is characterized by an imbalance between two COX metabolites of AA, namely, prostacyclin and especially thromboxane (TX). This imbalance of the biological actions of these two eicosanoids explains the major clinical symptoms of PE, such as hypertension, platelet aggregation, and reduced uteroplacental blood flow. Platelet TX synthesis is only increased in severe PE cases, additionally the proinflammatory cytokines such as IL-6 and TNFα are secreted excessively by maternal immune cells in preeclampsia [2]. In the placenta and leukocytes, the imbalance is exacerbated by the increased production of TX, coupled with the decreased production of prostacyclin in both mild and severe PE cases [78]. In the prevention of PE, a low dose of aspirin (50–150 mg/day) was considered because it selectively inhibits TX synthesis. Several studies have seen a drastic decrease in the incidence of PE after low-dose aspirin therapy [78].

The Gyselaers review indicates the multidimensionality of PE pathophysiology. PE seems to be caused by a complex cascade of events caused by abnormalities of both mother and child. The author points to factors related to the fetal adaptation process, followed by the mother’s hemodynamics and abnormalities related to the mother’s immune system and finally to communication disorders between the pregnant woman and the fetus [79]. Obesity is an important risk factor for PE. There is a positive correlation between BMI and the severity of PE, as well as the incidence of perinatal death [80]. Physiological pregnancy leads to strong uterine vessel remodeling, which allows increased blood flow to the placenta.

Trophoblast cells are stimulated to invade the maternal decidua and myometrium and reach the spiral arteries. The trophoblast migration from the placenta into uterine spiral arteries ends by transforming them into large channels with a lack of vasoconstrictive properties. In pathological pregnancies, this process is incomplete. The conversion of spiral arteries into high-capacity, low-resistance vessels increases blood flow to the placenta, ensuring normal fetal growth [81,82]. Proper hemodynamics in the mother’s body in the initial process of implantation determines the proper course of vascular tension and their proper expansion. Abnormalities in the process of spiral arteries reconstruction lead to insufficient oxygenation of the placenta and, as a consequence, local oxidative stress, which in subsequent stages of pregnancy adversely affects the cardiovascular system [79].

PE clinical manifestations are preceded by abnormal uterine spiral artery remodeling during early pregnancy. In physiology, the implantation process involves trophoblast penetration through the endothelium and the invasion of the blood vessels up to the myometrial layer. Impaired and insufficient remodeling of spiral artery walls may favor the occurrence of various disorders, directly leading to ischemia and placental hypoxia [83,84]. As a consequence of abnormal placental development, this results in fetal growth restriction [80,85]. Fetal weight is negatively correlated with maternal plasma AA, but not DHA [86]. A high level of arachidonic acid (study on mice) during gestation is associated with fetal growth restriction (FGR) through placental oxidative stress, with females being more susceptible to higher lipid and protein oxidation compared to a control group [86]. It is also suspected that an excess of dietary PUFAs may also enhance peroxidation and reduce antioxidant capacity, thereby influencing FGR [87].

In pregnancies complicated by PE, a significant expression of 15-LOX-1 and 15-LOX-2 has been demonstrated in placental tissues and umbilical arteries when compared to a control group. In addition, in PE pregnancies, an increased constriction of human umbilical artery (HUA) rings has been demonstrated to be dependent on 15-HETE levels. For this, 15-HETE increases HUA ring tension by increasing intercellular calcium levels [88]. In turn, Yuan et al. proved that in PE complicated pregnancies the placental 15-HETE is overly synthesized (15-HETE was found to be present in venous and umbilical cord blood as well) [89]. The study has also shown HIF-1α/15-LOX/15-HETE axis activation, which induces an abnormal endothelial cell migration process [89].

These factors may alter endothelial cell function, resulting in vasoconstriction, microangiopathy, increased blood pressure, and renal hypoperfusion. Based on the knowledge that 20-HETE causes renal arterial constriction and affects renal autoregulation, one would expect an increase in 20-HETE in pregnancies at risk of PE [90]. In a cross-sectional study conducted by Jiang et al. [85], in 69 women (19 pregnant women with PE, 29 pregnant women with normal blood pressure, and 21 nonpregnant women as a control group), no significant differences in plasma 20-HETE levels were found between the analyzed groups, however, the 20-HETE levels were shown to be higher in umbilical cord blood when compared to peripheral blood plasma [85].

The role of 20-HETE in blood pressure regulation is complex. It has a hypertensive activity by the promotion of constriction and vascular dysfunction and hypotensive activity by inhibiting renal salt reabsorption. For this, 20-HETE prevents the activation of calcium-activated potassium channels as well. In physiology, KCa activation is accompanied by an increase in the intracellular calcium concentration in the blood vessels of smooth muscle cells [91]. In vascular systems, the activity strongly constricts blood vessels and promotes the occurrence of hypertension. In addition, 20-HETE has been found to increase oxidative stress via activation of the production of super oxides and other reactive oxygen species (ROS) [92].

In the adrenal medulla, 20-HETE induces urinary sodium excretion [93]. This may affect natriuresis and contribute to the drop in blood pressure observed in the second trimester of pregnancy [8]. Abnormalities associated with 20-HETE metabolism may contribute to the development of vascular diseases, e.g., arterial hypertension by inducing endothelial and smooth muscle cell disorders [83]. Additionally, 20-HETE inhibits ion transport in nephrons and renal arteriole constriction. Nitric oxide (NO) has been shown to inhibit CYP4A expression, as well as the synthesis and activity of 20-HETE. NO has been shown to bind to isoforms of the CYP-450 enzyme, i.e., CYP4A1 and CYP4A3, and inhibit 20-HETE synthesis by inhibiting catalytic activity in female rats. This means that NO is a buffer with an anti-vasoconstrictor effect and presents an opposing action to 20-HETE [94].

The studies by Llinás et al. have demonstrated a reduction in 20-HETE levels in rats (from renal cortex tissue) with uterine ischemia and PE features due to cytochrome p450 activity inhibition, which thereby stimulates 20-HETE formation. In addition, in rats, the inhibition of cytochrome p450 activity causes a reduction in placental perfusion pressure, which in turn reduces hypertension. This suggests the involvement of 20-HETE in the induction of hypertension and renal vasoconstriction in pregnant rats with chronic uterine perfusion pressure [90]. The 20-HETE to EET ratio in rats with PE is increased. In addition, the prohypertensive effect of 20-HETE has been observed in a rat model group [93]. In contrast, the administration of the 20-HETE inhibitor (HET0016) resulted in the inhibition of blood vessels formed by 20-HETE. This inhibitor induces vasoconstriction reduction and improves uterine artery resistance [93]. In a study by Plenty et al. [81] blood and placenta samples were collected from PE-complicated pregnant women who qualified for a cesarean section. For this, 20-HETE production in microsomes isolated from the placenta were significantly increased in PE women compared to the control group. In addition, an increase in 12-HETE production was also observed in women with PE compared to the controls. However, no significant increase in 15-HETE was noted in the group of women with PE and neither was any significant statistical difference in the 20-HETE level observed in the blood. Significantly lower EET levels in the plasma of PE women compared to the control group were observed. In addition, the ratio of 20-HETE to EETs was significantly higher in women with PE. Furthermore, a lower concentration of 12-HETE was observed in women with PE compared to the control group in relation to the placental trophoblast cells. To summarize, the authors of the study found significantly higher HETE production in women with PE. Higher 15-HETE synthesis in trophoblasts isolated from the placentas of women with preeclampsia, in relation to physiological pregnancy, was observed by Johnson et al. [95]. In the Pearson et al. [96] studies analyzing myometrial biopsy specimens taken during cesarean sections it was shown that the presence of 5- and 6-EET was associated with uterine relaxation and affected vasodilatation as well. In contrast, 12-HETE and 20-HETE were associated with arterial uterine vasoconstriction [97].

In the placentas of women with PE, significantly higher 8- and 12-HETE and 8- and 9-EET concentrations were also found when compared to physiological pregnancies. The authors suggest that changes in the EET and HETE concentrations may result in poor placental perfusion, which is likely to contribute to the PE pathogenesis [98]. Interestingly, EET acids have a protective effect on blood vessels by acting as hyperpolarizing agents derived from the endothelium (they are produced by the amnion, chorion, placenta, decidual tissue, and myometrium), thereby lowering the impact of blood pressure [85]. In addition, urinary EET excretion was reduced and the 20-HETE blood levels remained unchanged in women with PE [85]. In another study with pregnant women at a 20-week gestation period, higher 11-, 12-EET, 5-HETE, 8-HETE, 12-HETE, and 15-HETE concentrations were found in PE-complicated pregnancies compared to normal pregnancies and nonpregnant women. Moreover, women with severe preeclampsia had significantly higher 5-HETE and 15-HETE serum levels compared to women with mild PE, as well as those of physiological pregnancies and nonpregnant women [99]. In terms of pregnancy pathologies, an interesting observation was made for the CYP11A1 isoform, which is an enzyme whose overexpression is present in PE cases and induces a higher level of cholesterol-synthetized pregnenolone and a decrease in trophoblast proliferation [100]. The described relationships are shown in Figure 3. It seems that gestational diabetes, like preeclampsia, activates the same signaling pathways, enhancing the synthesis of AA metabolites by LOX and CYP.

## 4. Summary

AA and LA derivatives play important roles in diseases affecting fertility and pregnancy pathologies. Although their roles are still insufficiently understood and require further research, it seems that HODE and HETE (especially in women affected by being overweight or obese presenting a greater risk for PCOS) could serve as a predictive marker in pregnancy pathologies, such as gestational diabetes mellitus or preeclampsia. Moreover, a male semen defect was found to be associated with simultaneous excessive PUFA increase, including AA in sperm, which promotes the excessive production of ROS and lipid peroxidation. Higher levels of n-6 in sera were correlated with reduced sperm motility, as well as reduced sperm count, and metabolites from the LOX pathway stimulate steroidogenesis. Before the investigational inhibitors or analogs of proresolving mediators are applied in various pathological conditions associated with reproduction and pregnancy, the relationship between FFA levels and their derivatives should be carefully tested in well-organized additional research studies, since there is insufficient literature on this topic.

## 5. Data Search Algorithm

The present review evaluates the above-mentioned topics by considering the literature published up to the 29 February 2020. A literature search was conducted utilizing the PubMed and Embase (Elsevier) databases. 

**Figure 4:**
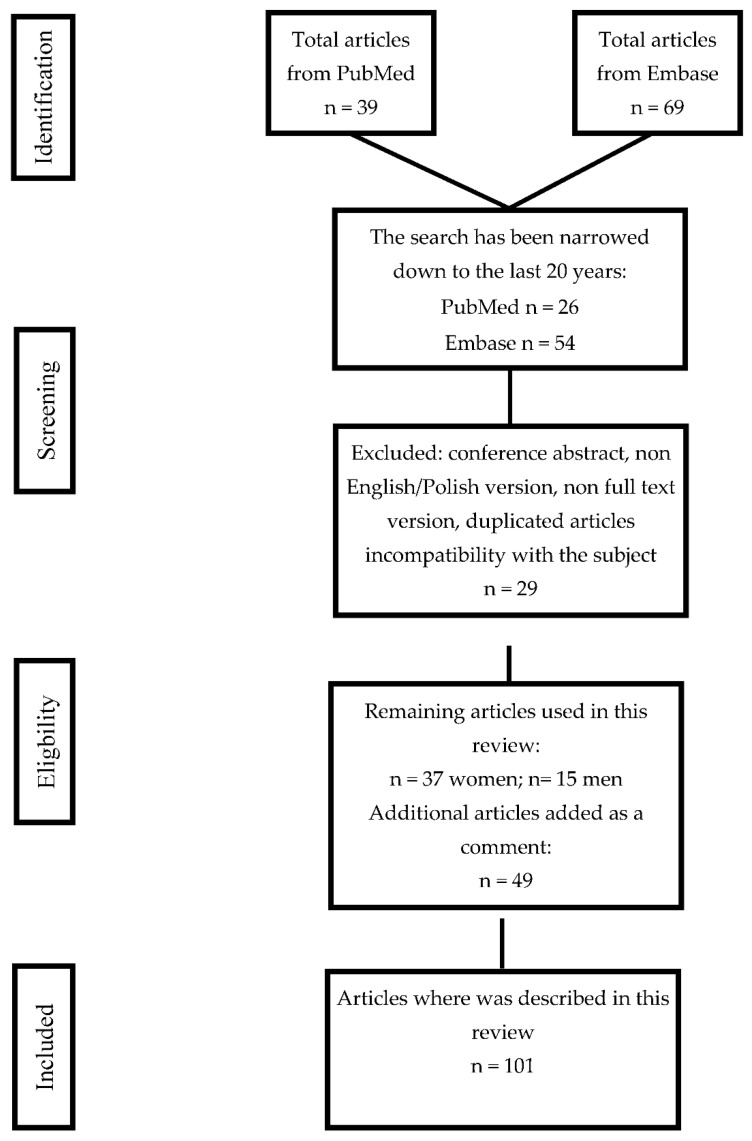
Flow diagram. Studies included in the review.

Search strategy: the present study reviewed papers that focused on pregnancy disorders and the arachidonic acid derivatives HETE and HODE by searching both database records from the past 20 years. All articles collected in the electronic search process and those used in this article have been reviewed. Database records not connected with the topic, as well as duplicated articles in both PubMed and Embase and conference abstracts, non-English versions, etc., were excluded from the review process. Studies which have investigated the association between pregnancy and arachidonic acid derivatives were included in our review. The current research was performed using the combinations of following keywords: Pregnancy disorders + “HETE” or “HODE” and “endometriosis” + “HETE” or “HODE” and “PCOS” + “HETE” or “HODE” and preeclampsia + “HETE” or “HODE” and gestational diabetes mellitus + “HETE” or “HODE”. At the end of the password “HODE” or “HETE” connected to the semen, sperm, spermatozoid. The supplementary keywords were arachidonic acid, linoleic acid, obesity, diabetes mellitus, and fetal growth restriction. In cases of duplicated information in the studies, the key selection was based on the highest contribution for the given topic. The search strategy scheme is shown below (Figure 4).
